# Using Smartphone-Tracked Behavioral Markers to Recognize Depression and Anxiety Symptoms: Cross-Sectional Digital Phenotyping Study

**DOI:** 10.2196/80765

**Published:** 2026-01-26

**Authors:** George Aalbers, Andrea Costanzo, Raj Jagesar, Femke Lamers, Martien J H Kas, Brenda W J H Penninx

**Affiliations:** 1 Department of Psychiatry, Amsterdam University Medical Center, Vrije Universiteit, Oldenaller 1, Amsterdam, 1081HJ, The Netherlands, 31 20 788 4666; 2 Mental Health Program, Amsterdam Public Health Research Institute, Amsterdam, The Netherlands; 3 Groningen Institute for Evolutionary Life Sciences Faculty of Science and Engineering, University of Groningen, Groningen, The Netherlands

**Keywords:** mobile health, mobile phone, digital phenotype, digital biomarker, machine learning

## Abstract

**Background:**

Depression and anxiety are prevalent but commonly missed and misdiagnosed, an important concern because many patients do not experience spontaneous recovery, and the duration of untreated illness is associated with worse outcomes.

**Objective:**

This study aims to explore the potential of using smartphone-tracked behavioral markers to support diagnostics and improve recognition of these disorders.

**Methods:**

We used the dedicated Behapp digital phenotyping platform to passively track location and app usage in 217 individuals, comprising symptomatic (n=109; depression/anxiety diagnosis or symptoms) and asymptomatic individuals (n=108; no diagnosis/symptoms). After quantifying 46 behavioral markers (eg, % time at home), we applied a machine learning approach to (1) determine which markers are relevant for depression/anxiety recognition and (2) develop and evaluate diagnostic prediction models for doing so.

**Results:**

Our analysis identifies the total number of GPS-based trajectories as a potential marker of depression/anxiety, where individuals with fewer trajectories are more likely to be symptomatic. Models using this feature in combination with demographics or in isolation outperformed demographics-only models (area under the receiver operating characteristic curve*_Mdn_*=0.60 vs 0.60 vs 0.51).

**Conclusions:**

Collectively, these findings indicate that smartphone-tracked behavioral markers have limited discriminant ability in our study but potential to support future depression/anxiety diagnostics.

## Introduction

Depression and anxiety disorders commonly are not recognized by general practitioners (eg, for depression, sensitivity=47.3%‐50.1%; [1]). In practice, this diagnostic issue is an important concern because many patients do not experience (short-term) spontaneous recovery [2,3], and the duration of untreated illness is associated with worse outcomes [4]. Mounting evidence suggests depression/anxiety recognition might be improved by diagnostic prediction models that rely on smartphone-tracked behavioral markers such as homestay and app use (eg, for depression, sensitivity=72.5%‐75.0%) [5]. However, this research area—here referred to as digital phenotyping—is considered to be in its infancy [6]. More work is required to identify informative behavioral markers and evaluate their potential diagnostic utility for health care professionals.

Digital phenotyping refers to “moment-by-moment quantification of the individual-level human phenotype in situ using data from personal digital devices, in particular smartphones” [7]. By accurately and unobtrusively capturing mental illness dimensions in daily life (eg, sleep, social behavior), digital phenotyping could contribute to more precise disease stratification in the long run (ie, deep phenotyping [8]). However, an important first step is to evaluate if digital phenotyping can help us broadly distinguish individuals with and without depression/anxiety, above and beyond demographic features known to predict these symptoms (eg, age, sex, and years of education [9]). We here consider the potential use of smartphone-tracked location and app use for depression/anxiety recognition; other digital phenotyping data sources (eg, Bluetooth, accelerometer, light sensor) are beyond the scope of this article.

Theoretically, digital phenotyping should (to some extent) help distinguish symptomatic from asymptomatic individuals. By using the smartphone to continuously log individuals’ GPS-based location and phone use, we can quantify smartphone-tracked behavioral markers such as time spent at home [10-13] that overlap or correlate with psychopathological symptoms [14]. For instance, specific anxiety disorders (eg, agoraphobia, social phobia) are defined by avoidance of specific contexts, and therefore, we might reasonably expect individuals with these symptoms to spend less time at leisure places and more time at home [15]. Similarly, smartphone app use might be relevant as it captures information about a person’s social activity (eg, time spent on communication apps such as WhatsApp) [16] and sleep patterns [17,18], both of which are altered in depression/anxiety.

In the past decade, digital phenotyping research has provided evidence that passively logged location and smartphone use might be promising for depression/anxiety recognition. One relatively stable finding in the domain is that depression and anxiety are related to reduced locational variability (eg, lower variance and entropy, lower number of places visited, longer homestay) [14,19-22]. Research further suggests smartphone log data might contain diagnostically useful features [23]. For instance, some—although limited—evidence indicates depression might be indicated by greater duration [19,23] and entropy of smartphone use [24], and that increased social media and communication app use might predict momentary subjective stress [25]. Collectively, evidence indicates digital phenotyping data might have diagnostic utility.

An important limitation of digital phenotyping research remains that applications in clinical samples are relatively uncommon [26]. This is not surprising because these are more costly and difficult to investigate than convenience samples, but it is problematic because the envisioned use case of digital phenotyping is clinical [27]. The unique contribution of our study is that we analyze a sample of individuals with (n=109; symptomatic group) and without clinically relevant depression/anxiety symptoms (n=108; asymptomatic group) in whom up to 43 days of digital phenotyping data were collected with the Behapp platform [12,16,28,29] in the Netherlands Study of Depression and Anxiety (NESDA) [30].

Using an explainable artificial intelligence (XAI) approach, which has growing popularity in the domain (Shapley additive explanations [SHAP] [31]; eg, [19]), we aim to (1) identify which behavioral markers are indicative of depression/anxiety and explore the strength and nature of this relation and (2) develop and evaluate machine learning (ML) models that use these markers to recognize depression/anxiety. Notably, as our sample size is limited, we develop and evaluate this model not for model deployment in clinical practice but rather as an exploration to inform future, larger studies. Where applicable, we report in line with the recently published TRIPOD-AI (Transparent Reporting of a Multivariable Prediction Model for Individual Prognosis or Diagnosis Plus Artificial Intelligence) guidelines [32].

## Methods

### Data

We here analyze data collected in the NESDA [30], as part of the Stress in Action consortium project [33]. This study uses the method of Penninx et al [30], and the method description partly reproduces their wording. NESDA participants were initially included for a baseline assessment with clinical interviews and surveys (2004‐2007) and assessed for the seventh time at the 15-year follow-up (2019‐2023). NESDA was designed to be representative of individuals with depressive and anxiety disorders in different health care settings and stages of the developmental history. Initially, participants were recruited from mental health care organizations, primary care, and the community setting. Participants were eligible if they were between 18 and 65 years; fluent in Dutch; and did not meet criteria for psychotic disorder, obsessive-compulsive disorder, bipolar disorder, or severe addiction disorder.

Specially trained clinical research staff conducted the composite international diagnostic interview [34] to determine if participants met *Diagnostic and Statistical Manual of Mental Disorders, Fourth Edition* (*DSM-IV*) criteria for depression and anxiety disorders, and participants completed a battery of self-report surveys using depression and anxiety symptom measures, including the Inventory of Depressive Symptomatology (IDS) [35] and Beck Anxiety Inventory [36]. A subset of NESDA participants installed a digital phenotyping app (Behapp, for more information see [11,28,29]) on their smartphone and provided the app permissions to continuously log their location (longitude and latitude) and smartphone app use (timestamps of when a specific app was opened and closed). On average, Behapp was activated on the day of the interview (SD 4 d), and self-report surveys were completed an average of 12 days before app activation.

### Participants

We enrolled a total of 405 participants in the NESDA digital phenotyping study, 343 of whom had both clinical and digital phenotyping data (n=62 without any digital phenotyping data, who were excluded). Because iOS disallows app logging and this is an essential data source for our study, we excluded individuals with this operating system (n=24). Further, to ensure digital phenotyping data quality, we excluded individuals with fewer than 7 days of both app and GPS-based location data (n=102). Hence, all analyses were conducted in 217 participants. For a more extensive description of missingness patterns and a demographic comparison between iOS and Android users, see Multimedia Appendix 1. The overall sample size was determined by feasibility constraints (ie, we included as many participants as possible and we retained those with sufficient available data) rather than sample size calculation. We applied the commonly used 80/20 train-test split to determine the sample size for model development (training) and evaluation (testing). Power analysis using powerROC [37] showed that our test set sample size (217 * 0.20 arriving at 43-44 participants) is sufficient to confirm an area under the receiver operating characteristic curve (AUROC)≥0.80 (prevalence of events in the test set=0.5, target width for estimated AUROC 95% CI <0.60).

### Ethical Considerations

The NESDA study, including its digital phenotyping substudy, was approved by the Amsterdam UMC medical ethical committee (reference number 2003‐183). All participants provided informed consent for both clinical assessment and digital phenotyping.

For participation in a face-to-face assessment wave, respondents received a €15 (US $17.53) gift certificate and reimbursement for travel expenses in appreciation of their time and cooperation. All data were collected and processed in compliance with the General Data Protection Regulation (GDPR). To ensure participant privacy, we present only statistical aggregates that do not contain any personally identifiable information.

### Outcome

*DSM-IV*–based diagnoses of depressive disorders (dysthymia and major depressive disorder [MDD]) and anxiety (social anxiety disorder, panic disorder with and without agoraphobia, agoraphobia, and generalized anxiety disorder) were established with the Composite International Diagnostic Interview (version 2.1 [34]), either in person or via phone call. Depression and anxiety symptom severity were assessed with the 30-item IDS [35] and BAI [36]. Outcome assessment was consistent across demographic groups. To evaluate if digital phenotyping data can broadly differentiate between individuals with and without symptoms, we combined these measures to form a binary outcome variable: symptomatic and asymptomatic. Symptomatic individuals had at least 1 depressive or anxiety disorder diagnosis in the past 6 months or an IDS or a BAI score exceeding thresholds specified in the survey manuals (IDS>13, BAI>9). Asymptomatic individuals did not have a diagnosis, and both IDS and BAI scores were below this threshold.

### Predictors

We used the digital phenotyping platform Behapp to passively collect smartphone-based data without storing any content of web queries, messages, or calls, in compliance with the GDPR [38]. The Behapp app has already been successfully used to investigate neuropsychiatric phenotypes [10,11,13,16] and to measure behavioral changes during the COVID-19 pandemic [12]. In this study, the collected raw data consisted of GPS-based location and foreground app usage data. We sampled the participants’ latitude and longitude at least every 10 minutes (with higher sampling frequencies during movement). For foreground app usage, we logged when an individual opened and closed a specific app. The Behapp itself is an app that runs in the background and is only accessed for setting up data collection and to restart the app when no data are being collected.

Using the Behapp feature extraction pipeline, these raw data were used to compute features (ie, measurable quantities), such as total phone usage in hours per day or the percentage of time spent at home. Table 1 provides a synthetic overview of these features. Prior to model building, we applied data-driven feature selection (see below) to all available features and trained models using only the selected features. Note that although the feature ‘app addiction’ captures information that we believe conceptually maps onto app addiction, it is unclear how it relates to validated addiction surveys or addiction diagnoses.

**Table 1.** Synthetic overview of Behapp digital phenotyping features.

Feature group and features		Definition	Example
Location			
Number of (unique) stay points		Number of (unique) stay points (average per day). A stay point is a location where participants stay within a range of 150 m for more than 30 min. In the count of the unique stay points, repeated visits of the same stay point (eg, office) count as 1 visit or can be smaller than 1 when individual has a single stay point for most of data collection.	Day 1: Home, work, gym Day 2: Home, work, café Day 3: Home, theater, park ... Number of stay points (total)=9 Number of stay points (average per day)=9/3=3 Number of unique stay points (total)=6 Number of unique stay points (average per day)=6/3=2
Time spent stationary		Time spent at stay points (in min).	Day 1: Home (10 h), work (8 h), gym (1 h) Day 2: Home (13 h), work (9 h), café (2 h) Time spent at stay points (average per day)=(10 + 8 + 1) + (13 + 9 + 2)/2 = 21.5
Home		Most frequently visited stay point of the top 3 stay points where most time was spent at night.	Apartment a: 16 h Apartment b: 9 h, 12 h, 10 h, 11 h Night club location: 7 h Apartment b=>Home
Trajectories		Each set of location data points in between stay points is saved separately as a trajectory if they contain a minimum of 20 data points (totaling at least 30 min).	Location 1 (2 h), travel (35 min), location 2 (3 h)=>trajectory identified Location 1 (2 h), travel (25 min), location 2 (3 h)=>trajectory not identified
App use			
App frequency		Number of times apps (in categories) were opened per day.	WhatsApp is categorized as a communication app and Instagram as a social media app Day 1: WhatsApp from 10:00 to 10:01, WhatsApp from 11:00 to 11:01, and Instagram from 11:30 to 11:40 Day 2: WhatsApp from 10:00 to 10:01 Number of times communication apps opened=3/2=1.5 Number of times social media apps opened=1/2=0.5
App frequency at night		Number of times apps (in categories) were opened at night (between 00:00 and 05:00).	—^a^
App duration		Duration (sum and mean) for which apps (in categories) were opened per day.	Duration (sum, average per day) communication apps = (2 + 1)/2 = 1.5 min Duration (mean, average per day) communication apps = (1 + 1)/2 = 1 min
App duration at night		Duration (sum and mean) for which apps (in categories) were opened at night (between 00:00 and 05:00).	—
App addiction		A value between 0 and 1 where 1 means that in each time interval of 20 min apps have been used at least once (average per day).	WhatsApp from 10:00 to 10:01 WhatsApp from 11:00 to 11:01 WhatsApp from 12:00 to 12:01 WhatsApp from 13:00 to 13:01 No other usage until 14:00 Addiction = 4/(4 * 3) = 0.33 4*3 represents the number of 20-min intervals between 10:00 and 14:00

a Not applicable.

### Analytical Methods

**Figure 1.**
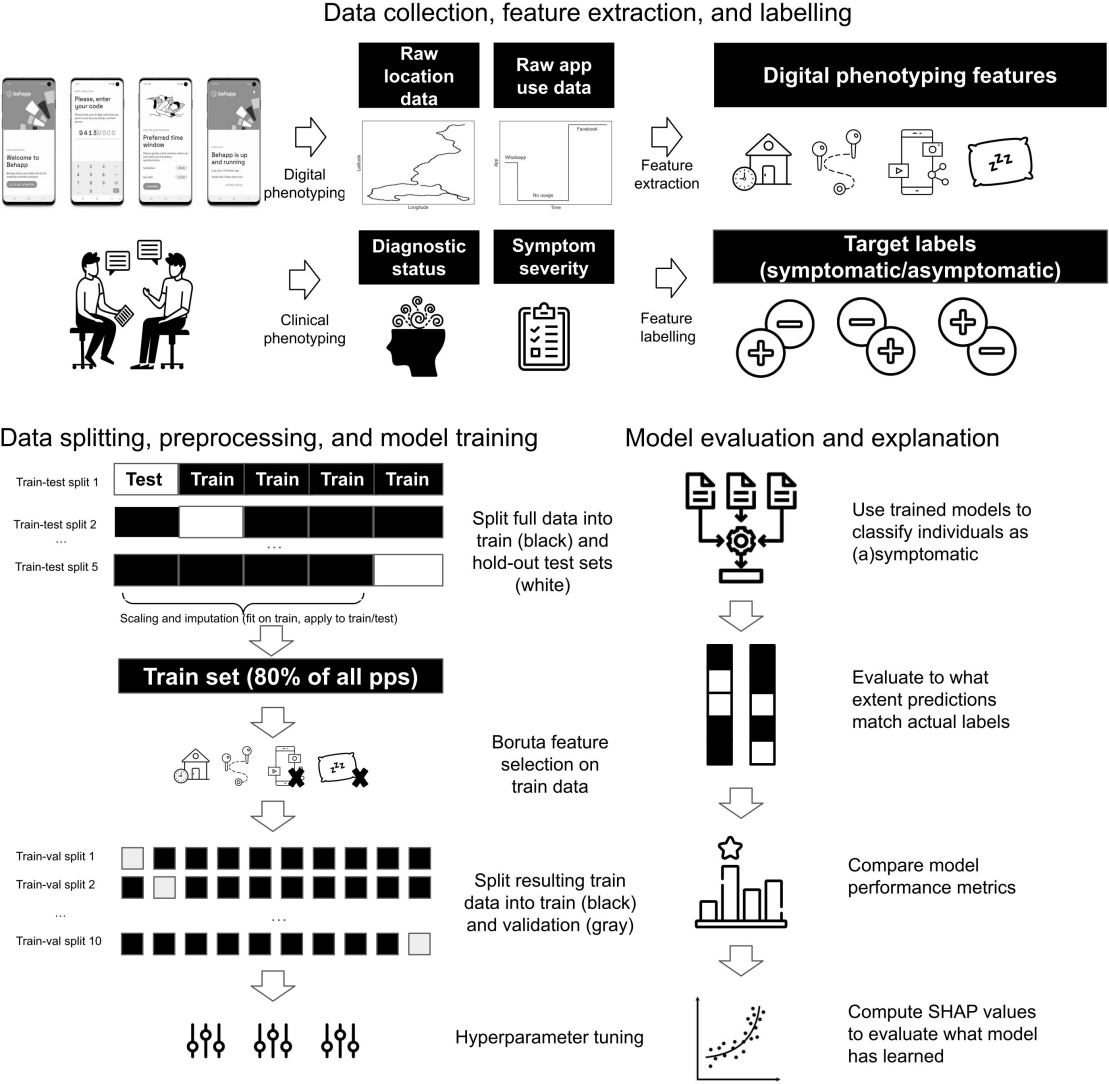
Visualization of the study design and machine learning pipeline. SHAP: Shapley additive explanations.

Figure 1 visualizes our ML pipeline (see [39] for all required Python code). After extracting features from the raw location and app usage data, we randomly split features and their corresponding psychiatric labels into 5 partitions, each containing (n=43) data points, corresponding to 20% of our total sample. To uniformly distribute individuals with and without depression/anxiety symptoms across data partitions, we apply a stratified 5-fold data split. We then iteratively select 4 partitions (referred to as the training set) and use these for minimum-maximum feature scaling, missing feature value imputation, feature selection, and hyperparameter tuning (with 10-fold stratified cross-validation) of a linear (ElasticNet logistic regression [LR]) and tree-based ML model (random forest [RF]) on each of 3 feature (sub)sets (ie, demographic, digital phenotyping, combined), after which we evaluate models on the remaining data partition (referred to as the test set). To impute missing feature values, we computed the mean of each feature in a training set and replaced the missing values in both this training set and its associated test set with this value. This procedure was repeated for all train-test set pairs. We repeat all steps until each data partition has been held out of model training once. Finally, for all trained models, we compute and visualize SHAP [31] values to clarify how models make their predictions based on specific feature values.

### Feature Selection

To reduce our initial feature set to a subset of potentially informative items, we applied the well-known Boruta feature selection algorithm [40], which selects an all-relevant subset of features by first reshuffling the original features into so-called “shadow features” and training RF classifiers to determine if the former are more informative about the target than the latter. The added value of RF, a tree-based ensemble model [41], over a traditional linear model is the capacity to learn discontinuous, interactive associations without making assumptions (eg, absence of multicollinearity) that are likely to be violated in digital phenotyping data (eg, multicollinearity due to feature similarity).

### Model Training

Using the Python library Scikit-learn [42] (version 1.5), we applied grid search stratified 10-fold cross-validation to tune hyperparameters of elasticNet-regularized LR [43] and RF [41] to maximize the model’s AUROC in validation data. We maximize the AUROC as this metric is typically used to assess how well a diagnostic prediction model can differentiate between individuals with and without a certain health outcome [32].

### Model Evaluation

To estimate how well models might differentiate between individuals with and without depression/anxiety symptoms in a real-world setting, we let trained models make predictions on the hold-out test data (ie, 20% patients) and then evaluate to what extent they can correctly classify individuals with and without depression/anxiety. We used Scikit-learn (version 1.5) to compute evaluation metrics applied in related work [19] (accuracy, AUROC, F1, precision, and recall, computing F1, precision, and recall separately for asymptomatic (F10, Precision0, Recall0) and symptomatic individuals (F11, Precision1, Recall1). Because we use 5-fold nested cross-validation—which means we train models on five train-test splits—we also evaluate each trained model on 5 hold-out test sets. For each trained model, we provide the median score for each evaluation metric. To determine which model performed best, we use the AUROC as our primary evaluation metric, as this is typically done for binary classification tasks [32].

For theory-driven researchers and clinicians, an important limitation of the RF classifier is that this model does not have the interpretable parameters that make up linear models. We therefore explain our models using the Python library SHAP (version 0.46.0) to compute and visualize SHAP [31] values as a beeswarm plot. A beeswarm plot visualizes how changes in feature values affect probabilities output by the model. This visualization might be thought of as a visual stand-in for parameter estimates in linear models.

## Results

### Descriptives

**Figure 2.**
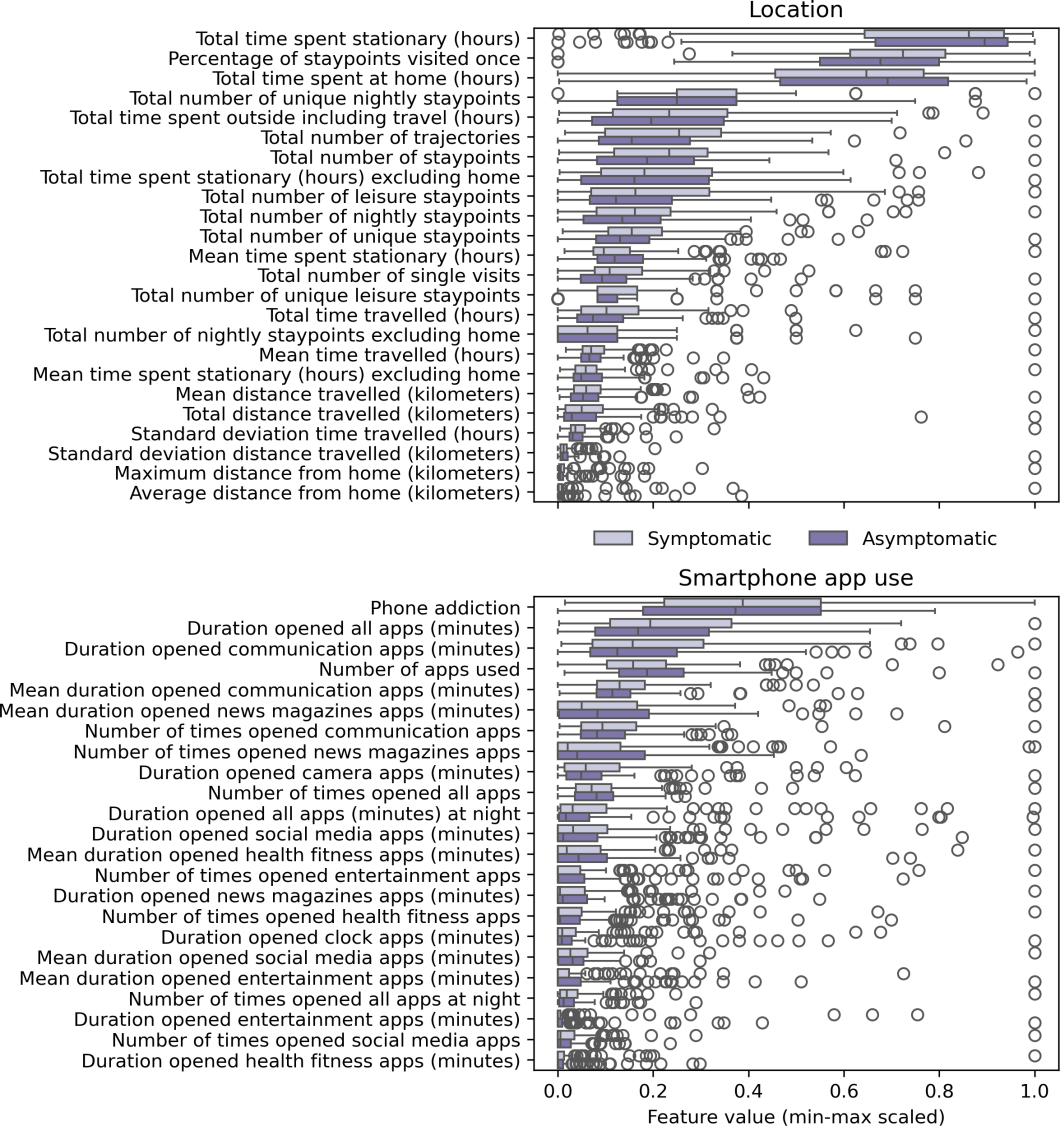
Multiboxplot representing distributions of minimum and maximum scaled digital phenotyping features for each group (symptomatic=dark purple and asymptomatic=light purple). Plots are categorized by feature group (upper panel location, lower panel smartphone app use) and sorted by overall feature mean.

Demographics were similar in the 2 groups, although female participants were overrepresented in the symptomatic group (Table 2). Relatively few individuals in the symptomatic group had a current diagnosis, with MDD being the most common diagnosis (n=26), followed by social phobia (n=16). However, by design, self-reported depression and anxiety symptoms were higher in the symptomatic than in the asymptomatic group. The median individual had 42 days of GPS-based location data and up to 43 days of app usage data. On average, symptomatic and asymptomatic individuals differed most strongly in their total number of leisure stay points, number of trajectories, duration of entertainment apps, and number of apps used (Multimedia Appendix 2). Most feature distributions were nonnormal, with the highest densities generally at the left tail. Feature distributions strongly overlap between symptomatic and asymptomatic individuals. Minor distributional differences are visible for features that quantify locational variability (ie, total time spent stationary, percentage of stay points visited once, total number of trajectories, total number of stay points, mean time spent stationary, total time traveled), and communication app use (Figure 2).

**Table 2.** Demographic and clinical descriptives for the asymptomatic and symptomatic groups.

Domain and Variable			Asymptomatic (n=108)	Symptomatic (n=109)
Demographics				
Age (years), mean (SD)			55.08 (12.82)	53.39 (12.24)
Years of education, mean (SD)			13.86 (2.88)	13.27 (3.28)
Sex (female), n (%)			65 (60.19)	78 (70.64)
Diagnosis in past 6 mo, n (%)				
Agoraphobia			—^a^	5 (4.59)
Panic disorder			—	12 (11.01)
Generalized anxiety disorder			—	6 (5.50)
Social phobia			—	16 (14.68)
Dysthymia			—	6 (5.50)
MDD^b^			—	26 (23.85)
Symptom severity, mean (SD)				
IDS^c^ total			6.22 (3.59)	21.63 (8.40)
BAI^d^ total			2.84 (2.50)	11.00 (6.35)
Data availability (days), median (IQR; range; SD)				
Location			42.00 (25.75-43; 7-43; 11.53)	42.00 (30-43; 7-43; 10.09)
App usage			42.50 (25-43; 8-43; 11.77)	43.00 (32-43; 8-43; 10.09)

a Not applicable.

b MDD: major depressive disorder.

c IDS: Inventory of Depressive Symptomatology.

d BAI: Beck Anxiety Inventory.

### Feature Selection

The total number of GPS-based trajectories was selected in all train-test splits. Other features, the majority of which were locational (n=7 vs n=3 app use), were generally selected in only 1 data split (eg, mean duration of communication app use). Therefore, with respect to the full dataset and relative to all the other features, a person’s number of location trajectories appears to most reliably indicate depression/anxiety. After an initial round of training and evaluating models, we observed suboptimal model performance, which we attributed to overfitting on feature selection. We therefore decided to retrain all models with the total number of GPS-based trajectories as the only digital phenotyping feature, as this was clearly the most stable feature of the data, and we successfully improved model performance by doing so. We present model performance and explanations of these simplified models here. Please note that model performance could be inflated due to decisions informed by the hold-out test data.

### Model Performance

To evaluate if features are predictive not only in the train but also in the hold-out test data, we trained 2 common ML model types (LR, RF) to recognize depression/anxiety from digital phenotyping and demographic data. We considered the predictive performance of models trained with only the total number of GPS-based trajectories as their feature and models using a combination of the total number of GPS-based trajectories and demographic features (age, sex, and years of education). We compared how well these models performed relative to two baseline models: a dummy model that uniformly outputs asymptomatic or symptomatic groups and models trained using only demographic features.

Considering median AUROC values as the primary metric, LR trained on only the total number of GPS-based trajectories performed best across train-test splits. AUROCs for this model (AUROC*_Mdn_*=0.61, Table 3) exceeded those of both baseline models (dummy model AUROC*_Mdn_*=0.50; LR demographics model AUROC*_Mdn_*=0.52). LR trained on the combined feature groups (AUROC*_Mdn_*=0.56) outperformed both baseline models but performed worse than the LR using GPS-based trajectories. Performance of RF models trained on combined feature groups (AUROC*_Mdn_*=0.60) was equal to that of RF models trained on GPS-based trajectories (AUROC*_Mdn_*=0.60) and better than both baselines (dummy model AUROC*_Mdn_*=0.50; RF demographics model AUROC*_Mdn_*=0.51).

**Table 3.** Model performance metrics (median-aggregated across hold-out test sets, range in parentheses).

Feature group and model	AUROC^a^	F10	F11	Precision0	Precision1	Recall0	Recall1	Accuracy
Baseline model, median (IQR; range)								
DM^b^	0.50 (0.50-0.50; 0.50-0.50)	0.47 (0.45-0.55; 0.45-0.56)	0.47 (0.45-0.52; 0.45-0.56)	0.45 (0.45-0.55; 0.45-0.55)	0.48 (0.45-0.52; 0.45-0.57)	0.48 (0.45-0.55; 0.45-0.57)	0.45 (0.45-0.52; 0.45-0.55)	0.47 (0.45-0.53; 0.45-0.56)
All, median (IQR; range)								
LR^c^	0.56 (0.51-0.65; 0.49-0.67)	0.49 (0.47-0.55; 0.46-0.63)	0.61 (0.57-0.61; 0.42-0.72)	0.58 (0.53-0.60; 0.45-0.75)	0.54 (0.52-0.58; 0.48-0.64)	0.48 (0.41-0.52; 0.41-0.55)	0.64 (0.64-0.71; 0.45-0.82)	0.56 (0.52-0.58; 0.47-0.68)
RF^d^	0.60 (0.60-0.61; 0.58-0.64)	0.60 (0.55-0.62; 0.46-0.62)	0.54 (0.53-0.60; 0.46-0.63)	0.56 (0.56-0.61; 0.50-0.62)	0.58 (0.53-0.62; 0.53-0.62)	0.64 (0.62-0.64; 0.36-0.71)	0.50 (0.45-0.59; 0.41-0.76)	0.57 (0.56-0.58; 0.51-0.61)
Digital phenotyping, median (IQR; range)								
LR	0.61 (0.60-0.61; 0.56-0.62)	0.57 (0.56-0.62; 0.5-0.62)	0.59 (0.57-0.60; 0.46-0.64)	0.58 (0.53-0.61; 0.52-0.65)	0.58 (0.54-0.62; 0.53-0.63)	0.64 (0.50-0.64; 0.48-0.67)	0.59 (0.55-0.59; 0.41-0.71)	0.61 (0.53-0.61; 0.52-0.61)
RF	0.60 (0.58-0.62; 0.52-0.62)	0.59 (0.58-0.61; 0.43-0.62)	0.47 (0.45-0.53; 0.43-0.57)	0.52 (0.52-0.58; 0.43-0.58)	0.56 (0.53-0.59; 0.45-0.60)	0.67 (0.64-0.68; 0.43-0.68)	0.45 (0.41-0.48; 0.36-0.55)	0.54 (0.52-0.58; 0.44-0.59)
Demographics, median (IQR; range)								
LR	0.52 (0.51-0.62; 0.48-0.63)	0.49 (0.46-0.51; 0.39-0.53)	0.60 (0.57-0.61; 0.56-0.70)	0.56 (0.53-0.60; 0.47-0.75)	0.54 (0.52-0.56; 0.50-0.59)	0.41 (0.41-0.41; 0.33-0.48)	0.64 (0.64-0.71; 0.64-0.86)	0.56 (0.52-0.56; 0.48-0.64)
RF	0.51 (0.51-0.55; 0.47-0.61)	0.50 (0.46-0.51; 0.40-0.52)	0.55 (0.51-0.57; 0.50-0.57)	0.53 (0.50-0.53; 0.44-0.55)	0.52 (0.50-0.54; 0.46-0.54)	0.48 (0.41-0.50; 0.36-0.52)	0.59 (0.55-0.59; 0.50-0.62)	0.51 (0.51-0.53; 0.45-0.55)

a AUROC: area under the receiver operating characteristic curve.

b DM: dummy model. Accuracy is balanced for any minor class imbalance.

c LR: logistic regression.

d RF: random forest.

### Model Explanation

**Figure 3.**
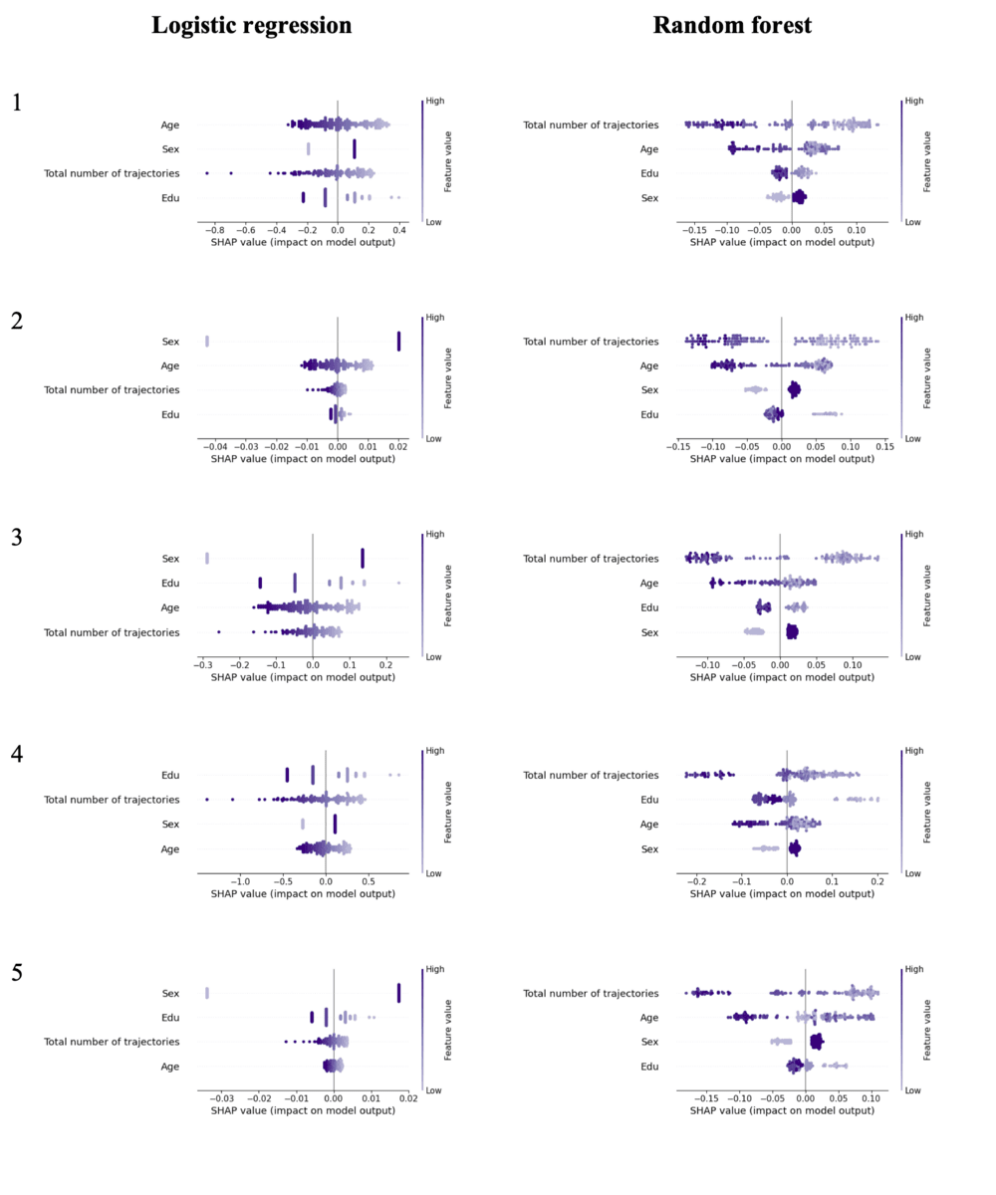
Beeswarm plots for the two model types trained using sex, age, years of education, and GPS-based number of trajectories as features. SHAP: Shapley additive explanations.

As nonlinear ML models such as RF do not have directly interpretable parameters, we computed and visualized SHAP values as beeswarm plots. By inspecting these plots, we learn how ML models transform feature values into probabilities, and in doing so, we gain insight into the mapping from features to the outcome that the models have learned. Figure 3 shows that LR and RF models consistently learned a negative relation between an individual’s total number of GPS-based trajectories per day and depression/anxiety. This means that for individuals with fewer trajectories, all models output a greater probability that they have depression/anxiety. Though capturing a relation with the same sign for this feature, LR and RF disagreed on its feature importance relative to demographic features. RF classifiers always assigned the highest feature importance to the total number of GPS-based locational trajectories, whereas LR always prioritized one or more demographic features.

## Discussion

### Principal Findings

To explore if digital phenotyping has the potential to support a diagnosis of depression/anxiety, we applied XAI to a unique combination of location, app use, and clinical assessment data collected in a subsample of NESDA participants with (n=109; symptomatic) and without depression/anxiety disorders or clinically relevant symptoms (n=108; asymptomatic). Our general findings suggest behavioral markers extracted from location and app use data potentially carry information about depression/anxiety, although their capacity to distinguish between those with and without clinically relevant symptoms is limited in the currently used data set.

Using a data-driven approach, we identify a number of GPS-based trajectories as a candidate behavioral marker for future studies. The total number of GPS-based trajectories, a locational feature that measures how frequently an individual moves between different stay points, was consistently selected across data splits, while other features were generally selected only once. Descriptive statistics and XAI analysis showed that individuals with fewer trajectories are more likely to be symptomatic and that this relation holds above and beyond demographic factors.

Our finding that individuals with fewer GPS-based trajectories are more likely to have depression/anxiety symptoms fits with previous empirical findings, providing more evidence that GPS-based behavioral features map onto depression/anxiety symptoms. A consistent finding in digital phenotyping has been that those with reduced locational variability (eg, lower variance and entropy, longer homestay) tend to have more depression and/or anxiety symptoms [19-22]. Conceptually, this behavioral feature fits with depression and anxiety symptoms that might diminish an individual’s tendency to approach rewarding experiences (eg, anhedonia) or might reinforce their tendency to avoid negative experiences (eg, specific locations or situations such as social situations). Such symptoms potentially might cause individuals to get stuck in places (or rather to prevent them from getting unstuck), which would manifest itself as reduced GPS-based trajectories. Changes in GPS-based trajectories might have clinical use in terms of monitoring symptoms, but could also point to an intervention opportunity where individuals are encouraged to increase their daily number of trajectories.

### Comparison With Prior Work

Model performance was less optimistic than in related ML work [19,23,24] but is consistent with statistically oriented studies that show weak relations between locational features and depression/anxiety [21,22]. Adequate study-to-study comparisons remain difficult to make, however, because digital phenotyping and clinical measures, sample characteristics, and modeling decisions differ from study to study and are likely to explain performance gaps. Hence, as suggested by others [44], an important avenue for digital phenotyping will be to harmonize study designs (eg, data collection, feature extraction, model types, cross-validation) to facilitate comparisons that are required to more adequately monitor progress in the domain. This call has been answered by academic consortia such as Stress in Action [33] that aim to collect digital phenotyping data at scale.

Model explanations using SHAP [31] showed our models have learned associations that are partially consistent with previous digital phenotyping studies. We found the total number of trajectories—which is conceptually similar to standard measures such as location variance or entropy—to be most important relative to all other Behapp features and negatively related to depression/anxiety, which matches previous findings [20,22,23,45] and well-known patterns that characterize depression/anxiety (eg, reduced motivation, social withdrawal [23]). However, contrary to previous work [24], our study did not identify any app use features as reliable predictors of depression/anxiety. Conceivably, this is because the relation between app use and depression/anxiety might hold in a specific subgroup only, as previous evidence suggests the association between app use and mental well-being potentially might differ from person to person [25,46-48].

Our findings are of interest not only for the development of diagnostic support systems but also for predict and preempt systems that aim to facilitate relapse prevention [49]. Diagnostic support systems aim to separate symptomatic from asymptomatic individuals based on differences between individuals (eg, symptomatic individuals tend to have fewer locational trajectories than asymptomatic individuals), while predict and preempt systems aim to identify onset of symptoms within an individual, based on behavioral differences between this individual’s asymptomatic and symptomatic periods (eg, when an individual’s trajectories start to decrease, they are increasingly symptomatic). Because our modeling approach, strictly speaking, is limited to between-subject conclusions, these findings do not necessarily imply that changes in the number of locational trajectories are indicative of symptom change. However, previous studies have already found within-person associations between locational features and depression/anxiety symptoms [21,22,50], indicating our findings might generalize from between-person to within-person and could potentially inform systems for relapse prevention.

### Limitations

An important contribution of our work is that it investigated the potential use of digital phenotyping in a sample that included individuals with a current disorder. This is still relatively uncommon in digital phenotyping [26] as clinical samples are more difficult to study than convenience samples. Notwithstanding, our findings should be considered in light of the following limitations. Though larger than the average study in the domain (N=217 vs N_Mean_=82) [51], our sample size was limited and had restricted demographics, in particular regarding age range. Combined with the fact that we excluded iOS users from our analysis, generalizability is limited to middle-aged Android users. However, considering the small demographic differences between Android and iOS users in our sample, this issue seems limited. Android users, on average, were somewhat older than iOS users, but did not differ in years of education and gender. Recent work in larger samples, however, has shown that Android ownership predicts lower levels of education, income, and extraversion [52], while other evidence suggests Android users are more likely to be men and older [53]. Future digital phenotyping work is needed for both iOS and Android users, as digital phenotyping screening tools ideally would be deployed irrespective of an individual’s operating system (OS). Because technical architecture and privacy frameworks for a given OS might prevent certain data sources from being collected (eg, app use in iOS), this means digital phenotyping screening tools might need to be developed for each OS separately.

It is also important to note that—within the feasibility constraints on sample size that are very common in a clinical setting—we were unable to evaluate how digital phenotyping features relate to specific depression and anxiety disorder diagnoses. The sample we analyzed contained a limited number of individuals with current depression and anxiety diagnoses. However, because many individuals who experience substantial residual symptoms would be unsuitable to be included in a control group, we took this into account by combining participant diagnosis and symptom self-report. Further, we know from NESDA research reports that comorbidity between depression and anxiety disorders is high, especially when looking at lifetime prevalence. We have observed that about 60% to 80% of NESDA respondents have had depression and anxiety diagnoses [54] and, therefore, decided not to analyze them separately. However, symptom heterogeneity in our sample might have attenuated our ability to detect features that are relevant to specific disorders, such as social anxiety disorder. In a more homogeneous sample, other behavioral markers might be found to be relevant and, given that these behavioral markers could arguably map better onto specific symptom profiles, it is thinkable that model performance would be improved. We therefore encourage future work to consider comparisons of symptomatically homogeneous groups. Notwithstanding, research on heterogeneous samples such as our own is necessary to detect transdiagnostic smartphone-tracked behavioral markers.

Finally, we acknowledge that model performance might have been inflated as a result of data leakage. In an initial exploratory round of model evaluation on hold-out test data, we discovered model performance to be unstable across test sets (Multimedia Appendix 3). We attributed this to the potential overfitting feature selection in the individual training sets, which is a risk in small sample sizes. To stabilize model performance, we decided to only develop and evaluate models with the most consistently selected feature (ie, total number of GPS trajectories). Even though these generalized more reliably to hold-out test sets within our sample, it could be that the total number of GPS trajectories was consistently selected by chance and that our post hoc decision to only retain this feature might be tantamount to overfitting. Further, post hoc power calculations using powerROC [37] showed that, with the model performance in our study, our test sets were about three times smaller than what would be required to convincingly show models perform better than random guessing. It is therefore not surprising that follow-up DeLong model comparison tests showed no statistically significant differences in model performance nor that model evaluation metrics confidence intervals consistently overlapped with chance (Multimedia Appendix 4). All in all, these findings should be interpreted with caution and viewed as a first step that can inform larger-scale follow-up studies.

### Future Research Directions

We recommend the following for future work that aims to develop a digital phenotyping-based symptom recognition system that can adequately differentiate between symptomatic and asymptomatic individuals. To ensure digital markers are consistently defined across studies, digital phenotyping studies would benefit from developing and adhering to an ontology of digital markers (for an example under development, see [55]). This ontology would ideally map digital markers (and configurations thereof) to specific symptoms or syndromes and could still include behavioral markers that were not marked as relevant in the present dataset, but have a strong conceptual mapping onto disorder definitions (eg, homestay is a clinical marker of agoraphobia). In a sufficiently large dataset with adequate diagnostic labels (conceivably in the order of thousands of participants), such an ontology could be used to develop multigroup classification models that can leverage digital markers to identify individuals as having no symptoms or (symptoms of) one or more specific disorders (eg, agoraphobia or agoraphobia with MDD).

It is highly recommended to design future digital phenotyping studies with model evaluation in mind, using a prior power analysis. Given the model performance in this study, post hoc sample size calculation suggests that at least 122 individuals should be held out of training for model evaluation, meaning that a sample size of over 600 individuals would have been needed for both training and evaluation. Of note, fewer individuals would be required for sufficient statistical power if model performance is improved substantially, which might possibly be achieved with greater symptom contrasts between groups (ie, comparing individuals without symptoms to individuals with severe symptoms) and greater symptom homogeneity within groups (ie, comparing individuals without symptoms to individuals with a specific disorder).

Development of a multimodal digital phenotyping toolkit, longitudinal measurement of much larger samples, and follow-up research on theoretically relevant markers is underway in the Stress in Action consortium [33]. We envision that, over time, this multimodal digital phenotyping toolkit might be used to trigger traditional symptom screening instruments such as the 9-item Patient Health Questionnaire (PHQ-9) when symptoms are most likely (for a similar approach with wearables, see [49]), given changes in an individual’s digital phenotyping data. Symptom screening surveys have high sensitivity for detecting mental illness. For instance, the PHQ-9 has a sensitivity of 0.88 (95% CI 0.83 to 0.92) and a specificity of 0.85 (0.82-0.88) for MDD [56], which is unlikely to be outmatched by digital phenotyping models. In practice, however, individuals are unlikely to consistently complete symptom surveys for extended periods, which can be a significant burden. By using digital phenotyping, we might be able to help reduce this burden and potentially improve early symptom detection.

### Conclusion

In all, digital phenotyping, here operationalized as passive logging of location and app use, offers insights into behavioral patterns that could potentially differentiate individuals with clinically relevant depression/anxiety symptoms from those without. In the unique NESDA sample comprising both symptomatic and asymptomatic individuals, we identified a specific smartphone-tracked behavioral marker, namely the total number of GPS-based trajectories, that may indicate these symptoms. Our findings align with previous studies suggesting ML models might be able to leverage smartphone-tracked behavioral markers to recognize symptomatic individuals. Although we show that such markers cannot support diagnostics on their own, we believe they are sufficiently promising to be considered in future deep phenotyping of depression and anxiety.

## Supplementary material

10.2196/80765 Multimedia Appendix 1 Missingness patterns and operating system demographics.

10.2196/80765 Multimedia Appendix 2 Descriptives for the top 5 digital phenotyping features.

10.2196/80765 Multimedia Appendix 3 Model performance with initial exploratory Boruta feature selection.

10.2196/80765 Multimedia Appendix 4 Statistical model comparisons (DeLong test) and bootstrapped CIs.

## Abbreviations

AUROC: area under the receiver operating characteristic curve
BAI: Beck Anxiety Inventory
*DSM-IV*: *Diagnostic and Statistical Manual of Mental Disorders, Fourth Edition*
GDPR: General Data Protection Regulation
IDS: Inventory of Depressive Symptomatology
LR: logistic regression
MDD: major depressive disorder
ML: machine learning
NESDA: Netherlands Study of Depression and Anxiety
PHQ-9: 9-item Patient Health Questionnaire
RF: random forest
SHAP: Shapley additive explanations
TRIPOD-AI: Transparent Reporting of a Multivariable Prediction Model for Individual Prognosis or Diagnosis Plus Artificial Intelligence
XAI: explainable artificial intelligence

## References

[1] Mitchell AJ, Vaze A, Rao S (2009). Clinical diagnosis of depression in primary care: a meta-analysis. Lancet.

[2] Mekonen T, Ford S, Chan GCK, Hides L, Connor JP, Leung J (2022). What is the short-term remission rate for people with untreated depression? A systematic review and meta-analysis. J Affect Disord.

[3] Whiteford HA, Harris MG, McKeon G (2013). Estimating remission from untreated major depression: a systematic review and meta-analysis. Psychol Med.

[4] Ghio L, Gotelli S, Marcenaro M, Amore M, Natta W (2014). Duration of untreated illness and outcomes in unipolar depression: a systematic review and meta-analysis. J Affect Disord.

[5] Moura I, Teles A, Viana D, Marques J, Coutinho L, Silva F (2023). Digital phenotyping of mental health using multimodal sensing of multiple situations of interest: a systematic literature review. J Biomed Inform.

[6] Roefs A, Fried EI, Kindt M (2022). A new science of mental disorders: Using personalised, transdiagnostic, dynamical systems to understand, model, diagnose and treat psychopathology. Behav Res Ther.

[7] Onnela JP (2021). Opportunities and challenges in the collection and analysis of digital phenotyping data. Neuropsychopharmacology.

[8] Robinson PN (2012). Deep phenotyping for precision medicine. Hum Mutat.

[9] Licht CMM, de Geus EJC, Zitman FG, Hoogendijk WJG, van Dyck R, Penninx BWJH (2008). Association between major depressive disorder and heart rate variability in the Netherlands Study of Depression and Anxiety (NESDA). Arch Gen Psychiatry.

[10] Sverdlov O, Curcic J, Hannesdottir K (2021). A study of novel exploratory tools, digital technologies, and central nervous system biomarkers to characterize unipolar depression. Front Psychiatry.

[11] Jongs N, Jagesar R, van Haren NEM (2020). A framework for assessing neuropsychiatric phenotypes by using smartphone-based location data. Transl Psychiatry.

[12] Jagesar RR, Roozen MC, van der Heijden I (2021). Digital phenotyping and the COVID-19 pandemic: capturing behavioral change in patients with psychiatric disorders. Eur Neuropsychopharmacol.

[13] Kas MJH, Jongs N, Mennes M (2024). Digital behavioural signatures reveal trans-diagnostic clusters of schizophrenia and Alzheimer’s disease patients. Eur Neuropsychopharmacol.

[14] Wang R, Wang W, Dasilva A (2018). Tracking depression dynamics in college students using mobile phone and wearable sensing. Proc ACM Interact Mob Wearable Ubiquitous Technol.

[15] Boukhechba M, Chow P, Fua K, Teachman BA, Barnes LE (2018). Predicting social anxiety from global positioning system traces of college students: feasibility study. JMIR Ment Health.

[16] Muurling M, Reus LM, de Boer C (2022). Assessment of social behavior using a passive monitoring app in cognitively normal and cognitively impaired older adults: observational study. JMIR Aging.

[17] Aledavood T, Kivimäki I, Lehmann S, Saramäki J (2022). Quantifying daily rhythms with non-negative matrix factorization applied to mobile phone data. Sci Rep.

[18] Borger JN, Huber R, Ghosh A (2019). Capturing sleep-wake cycles by using day-to-day smartphone touchscreen interactions. NPJ Digit Med.

[19] Opoku Asare K, Moshe I, Terhorst Y (2022). Mood ratings and digital biomarkers from smartphone and wearable data differentiates and predicts depression status: A longitudinal data analysis. Pervasive Mob Comput.

[20] Moshe I, Terhorst Y, Opoku Asare K (2021). Predicting symptoms of depression and anxiety using smartphone and wearable data. Front Psychiatry.

[21] Chow PI, Fua K, Huang Y (2017). Using mobile sensing to test clinical models of depression, social anxiety, state affect, and social isolation among college students. J Med Internet Res.

[22] Zhang Y, Folarin AA, Sun S (2022). Longitudinal relationships between depressive symptom severity and phone-measured mobility: dynamic structural equation modeling study. JMIR Ment Health.

[23] Saeb S, Zhang M, Karr CJ (2015). Mobile phone sensor correlates of depressive symptom severity in daily-life behavior: an exploratory study. J Med Internet Res.

[24] Opoku Asare K, Terhorst Y, Vega J, Peltonen E, Lagerspetz E, Ferreira D (2021). Predicting depression from smartphone behavioral markers using machine learning methods, hyperparameter optimization, and feature importance analysis: exploratory study. JMIR Mhealth Uhealth.

[25] Aalbers G, Hendrickson AT, Vanden Abeele MM, Keijsers L (2023). Smartphone-tracked digital markers of momentary subjective stress in college students: idiographic machine learning analysis. JMIR Mhealth Uhealth.

[26] De Angel V, Lewis S, White K (2022). Digital health tools for the passive monitoring of depression: a systematic review of methods. NPJ Digit Med.

[27] Insel TR (2018). Digital phenotyping: a global tool for psychiatry. World Psychiatry.

[28] Behapp.

[29] Jagesar RR, Vorstman JA, Kas MJ (2021). Requirements and operational guidelines for secure and sustainable digital phenotyping: design and development study. J Med Internet Res.

[30] Penninx BWJH, Beekman ATF, Smit JH (2008). The Netherlands Study of Depression and Anxiety (NESDA): rationale, objectives and methods. Int J Methods Psychiatr Res.

[31] Lundberg SM, Lee SI A unified approach to interpreting model predictions.

[32] Collins GS, Moons KGM, Dhiman P (2024). TRIPOD+AI statement: updated guidance for reporting clinical prediction models that use regression or machine learning methods. BMJ.

[33] Stress in Action.

[34] Wittchen HU (1994). Reliability and validity studies of the WHO--Composite International Diagnostic Interview (CIDI): a critical review. J Psychiatr Res.

[35] Rush AJ, Gullion CM, Basco MR, Jarrett RB, Trivedi MH (1996). The Inventory of Depressive Symptomatology (IDS): psychometric properties. Psychol Med.

[36] Beck AT, Epstein N, Brown G, Steer RA (1988). An inventory for measuring clinical anxiety: psychometric properties. J Consult Clin Psychol.

[37] Grolleau F, Tibshirani R, Chen JH (2025). powerROC: an interactive WebwTool for sample size calculation in assessing models’ discriminative abilities. AMIA Jt Summits Transl Sci Proc.

[38] Mulder T, Jagesar RR, Klingenberg AM, P Mifsud Bonnici J, Kas MJ (2018). New European privacy regulation: assessing the impact for digital medicine innovations. Eur Psychiatry.

[39] George-aalbers/cross-sectional-digital-phenotyping-study. Github.

[40] Kursa MB, Rudnicki WR (2010). Feature selection with the Boruta package. J Stat Softw.

[41] Breiman L (2001). Random forests. Mach Learn.

[42] Pedregosa F, Varoquaux G, Gramfort A (2011). Scikit-learn: machine learning in Python. J Mach Learn Res.

[43] Zou H, Hastie T (2005). Regularization and variable selection via the elastic net. J Royal Stat Soc Series B.

[44] Rohani DA, Faurholt-Jepsen M, Kessing LV, Bardram JE (2018). Correlations between objective behavioral features collected from mobile and wearable devices and depressive mood symptoms in patients with affective disorders: systematic review. JMIR Mhealth Uhealth.

[45] Saeb S, Lattie EG, Schueller SM, Kording KP, Mohr DC (2016). The relationship between mobile phone location sensor data and depressive symptom severity. PeerJ.

[46] Aalbers G, vanden Abeele MMP, Hendrickson AT, de Marez L, Keijsers L (2022). Caught in the moment: are there person-specific associations between momentary procrastination and passively measured smartphone use?. Mobile Media & Communication.

[47] Verbeij T, Pouwels JL, Beyens I, Valkenburg PM (2022). Experience sampling self-reports of social media use have comparable predictive validity to digital trace measures. Sci Rep.

[48] Siebers T, Beyens I, Valkenburg PM (2024). The effects of fragmented and sticky smartphone use on distraction and task delay. Mob Med Commun.

[49] Vairavan S, Rashidisabet H, Li QS (2023). Personalized relapse prediction in patients with major depressive disorder using digital biomarkers. Sci Rep.

[50] Canzian L, Musolesi M Trajectories of depression: unobtrusive monitoring of depressive states by means of smartphone mobility traces analysis.

[51] Melcher J, Hays R, Torous J (2020). Digital phenotyping for mental health of college students: a clinical review. Evid Based Ment Health.

[52] Schoedel R, Reiter T, Krämer MD (2025). Person-related selection bias in mobile sensing research: robust findings from two panel studies. PsyArXiv.

[53] Shaw H, Ellis DA, Kendrick LR, Ziegler F, Wiseman R (2016). Predicting smartphone operating system from personality and individual differences. Cyberpsychol Behav Soc Netw.

[54] Lamers F, van Oppen P, Comijs HC (2011). Comorbidity patterns of anxiety and depressive disorders in a large cohort study. J Clin Psychiatry.

[55] Ontology for digital markers in mental health (ODIM-MH). GitHub.

[56] Levis B, Benedetti A, Thombs BD (2019). Accuracy of Patient Health Questionnaire-9 (PHQ-9) for screening to detect major depression: individual participant data meta-analysis. BMJ.

