# Correction: Using Smartphone-Tracked Behavioral Markers to Recognize Depression and Anxiety Symptoms: Cross-Sectional Digital Phenotyping Study

**DOI:** 10.2196/92888

**Published:** 2026-02-18

**Authors:** George Aalbers, Andrea Costanzo, Raj Jagesar, Femke Lamers, Martien J H Kas, Brenda W J H Penninx

**Affiliations:** 1 Department of Psychiatry, Amsterdam University Medical Center, Vrije Universiteit, Oldenaller 1, Amsterdam, 1081HJ, The Netherlands, 31 20 788 4666; 2 Mental Health Program, Amsterdam Public Health Research Institute, Amsterdam, The Netherlands; 3 Groningen Institute for Evolutionary Life Sciences Faculty of Science and Engineering, University of Groningen, Groningen, The Netherlands

In “Using Smartphone-Tracked Behavioral Markers to Recognize Depression and Anxiety Symptoms: Cross-Sectional Digital Phenotyping Study” [1], the authors noted one omission.

Author FL was originally linked to only affiliation 2. This has been amended so that they are linked to affiliations 1 and 2.

The correction will appear in the online version of the paper on the JMIR Publications website, together with the publication of this correction notice. Because this was made after submission to PubMed, PubMed Central, and other full-text repositories, the corrected article has also been resubmitted to those repositories.
