# Supplementary material for: Using Smartphone-Tracked Behavioral Markers to Recognize Depression and Anxiety Symptoms: Cross-Sectional Digital Phenotyping Study
Source: JMIR Ment Health. 2026 Jan 26;13:e80765. doi: 10.2196/80765 (PMC12836477; doi:10.2196/80765)
Supplement: Multimedia Appendix 1 [file mental-v13-e80765-s001.docx]

**Results from initial Boruta feature selection approach**

In a first exploratory step, we applied the Boruta feature selection algorithm to determine which digital phenotyping features are relevant for distinguishing between individuals with and without depression/anxiety symptoms, doing so for each of the five train sets. Because we applied Boruta to each of five (partially overlapping) train sets, this resulted in five (partially overlapping) sets of selected features (Table 1). We then trained models using the selected features in the pertinent train set (80% of all participants) and evaluated how well these models performed in the held-out test set (20% of all participants), computing the AUC ROC, F10, F11, Precision0, Precision1, Recall0, Recall1, and Accuracy (Table 2).

Table 1: Selected features in each train set sorted by the total number of times these were selected.

| Feature name | 1 | 2 | 3 | 4 | 5 | Total |
| --- | --- | --- | --- | --- | --- | --- |
| Total number of GPS trajectories | X | X | X | X | X | 5 |
| Number of apps used |  | X | X |  | X | 3 |
| Total time spent outside including travel hours | X | X |  | X |  | 3 |
| Mean time spent stationary |  |  | X | X | X | 3 |
| Mean time travelled |  |  |  | X |  | 1 |
| Duration opened all apps at night |  |  | X |  |  | 1 |
| Total time spent stationary |  |  |  | X |  | 1 |
| Mean duration opened communication apps |  |  |  | X |  | 1 |
| Standard deviation time travelled | X |  |  |  |  | 1 |
| Total distance travelled |  | X |  |  |  | 1 |
| Percentage of staypoints visited once |  | X |  |  |  | 1 |

Table 2 shows that out-of-sample performance of models with digital phenotyping features strongly varied from test set to test set, with AUC ROC values ranging from below 0.50 (i.e., lower than the dummy model) to approximately 0.6. Conceivably, such variability could be related to overfitting on feature selection in the train set, where Boruta feature selection retains features whose relevance is specific to one train set but does not generalize to a hold-out test set. To tackle this issue and select features that are at least relevant in all sections of our sample, we performed a two-step Boruta feature selection procedure, where we first applied Boruta as described above and then selected the features that were consistently selected across train sets. Only the total number of GPS trajectories met this criterion in our data and therefore we retrained and re-evaluated digital phenotyping-based models using only this feature.

Table 2: Model performance metrics (median, minimum, and maximum across hold-out test sets).

| Feature group | Model | AUC | | | F10 | | | F11 | | | Precision0 | | | Precision1 | | | Recall0 | | | Recall1 | | | Accuracy | | |
| --- | --- | --- | --- | --- | --- | --- | --- | --- | --- | --- | --- | --- | --- | --- | --- | --- | --- | --- | --- | --- | --- | --- | --- | --- | --- |
|  |  | Mdn | Min | Max | Mdn | Min | Max | Mdn | Min | Max | Mdn | Min | Max | Mdn | Min | Max | Mdn | Min | Max | Mdn | Min | Max | Mdn | Min | Max |
| All | DM | 0.5 | 0.5 | 0.5 | 0.47 | 0.45 | 0.56 | 0.47 | 0.45 | 0.56 | 0.45 | 0.45 | 0.55 | 0.48 | 0.45 | 0.57 | 0.48 | 0.45 | 0.57 | 0.45 | 0.45 | 0.55 | 0.47 | 0.45 | 0.56 |
|  | LR | 0.55 | 0.38 | 0.66 | 0.5 | 0.36 | 0.55 | 0.52 | 0.33 | 0.73 | 0.5 | 0.35 | 0.82 | 0.5 | 0.35 | 0.61 | 0.45 | 0.38 | 0.52 | 0.55 | 0.32 | 0.91 | 0.5 | 0.35 | 0.66 |
|  | RF | 0.54 | 0.4 | 0.61 | 0.5 | 0.45 | 0.61 | 0.54 | 0.37 | 0.55 | 0.5 | 0.44 | 0.56 | 0.5 | 0.44 | 0.61 | 0.57 | 0.41 | 0.67 | 0.5 | 0.32 | 0.59 | 0.5 | 0.44 | 0.58 |
| Digital phenotyping | LR | 0.58 | 0.37 | 0.6 | 0.54 | 0.49 | 0.57 | 0.59 | 0.43 | 0.65 | 0.57 | 0.48 | 0.67 | 0.56 | 0.48 | 0.59 | 0.5 | 0.45 | 0.67 | 0.59 | 0.36 | 0.76 | 0.57 | 0.48 | 0.61 |
|  | RF | 0.52 | 0.4 | 0.58 | 0.57 | 0.43 | 0.64 | 0.5 | 0.39 | 0.57 | 0.52 | 0.42 | 0.59 | 0.56 | 0.42 | 0.62 | 0.59 | 0.41 | 0.73 | 0.45 | 0.36 | 0.57 | 0.54 | 0.42 | 0.59 |
| Demographics | LR | 0.52 | 0.48 | 0.63 | 0.49 | 0.39 | 0.53 | 0.6 | 0.56 | 0.7 | 0.56 | 0.47 | 0.75 | 0.54 | 0.5 | 0.59 | 0.41 | 0.33 | 0.48 | 0.64 | 0.64 | 0.86 | 0.56 | 0.48 | 0.64 |
|  | RF | 0.51 | 0.47 | 0.61 | 0.5 | 0.4 | 0.52 | 0.55 | 0.5 | 0.57 | 0.53 | 0.44 | 0.55 | 0.52 | 0.46 | 0.54 | 0.48 | 0.36 | 0.52 | 0.59 | 0.5 | 0.62 | 0.51 | 0.45 | 0.55 |
