# Supplementary material for: Using Smartphone-Tracked Behavioral Markers to Recognize Depression and Anxiety Symptoms: Cross-Sectional Digital Phenotyping Study
Source: JMIR Ment Health. 2026 Jan 26;13:e80765. doi: 10.2196/80765 (PMC12836477; doi:10.2196/80765)
Supplement: Multimedia Appendix 2 [file mental-v13-e80765-s002.docx]

**Descriptives for the top 5 digital phenotyping features**

Table 1: Descriptives for the top 5 digital phenotyping features for discriminating between asymptomatic and symptomatic.

| Feature | M | Mdn | SD | M | Mdn | SD | d |
| --- | --- | --- | --- | --- | --- | --- | --- |
| **Application use features** |  |  |  |  |  |  |  |
| Duration entertainment apps (minutes, average per day) | 4.87 | 2.08 | 7.14 | 3.24 | 1.27 | 5.29 | 0.26 |
| Number of apps used (average per day) | 2.01 | 1.79 | 1.00 | 1.78 | 1.58 | 0.93 | 0.24 |
| Frequency communication apps (average per day) | 32.6 | 25.64 | 24.24 | 39.13 | 29.21 | 42.9 | 0.19 |
| Frequency all apps at night (average per day) | 4.36 | 1.94 | 6.78 | 6.65 | 3.00 | 16.06 | 0.19 |
| Frequency all apps (average per day) | 156.5 | 152.96 | 101.75 | 185.39 | 133.47 | 205.52 | 0.18 |
| **Location features** |  |  |  |  |  |  |  |
| Number of leisure staypoints (average per day) | 0.39 | 0.28 | 0.34 | 0.3 | 0.21 | 0.27 | 0.3 |
| Number of trajectories (average per day) | 1.29 | 1.37 | 0.79 | 1.09 | 0.84 | 0.89 | 0.24 |
| % of staypoints visited once (% of total number of staypoints) | 63.43 | 65.84 | 13.97 | 59.81 | 61.54 | 16.87 | 0.23 |
| Maximum distance from home (km, average per day) | 310.82 | 73.83 | 1110.5 | 140.19 | 54.64 | 296.15 | 0.21 |
| Nightly staypoints (average per day) | 0.07 | 0.06 | 0.06 | 0.06 | 0.05 | 0.04 | 0.21 |

*Note.* Features are sorted (descending and per feature group) by absolute Cohen’s d of the symptomatic vs asymptomatic group difference.
