# Supplementary material for: Using Smartphone-Tracked Behavioral Markers to Recognize Depression and Anxiety Symptoms: Cross-Sectional Digital Phenotyping Study
Source: JMIR Ment Health. 2026 Jan 26;13:e80765. doi: 10.2196/80765 (PMC12836477; doi:10.2196/80765)
Supplement: Multimedia Appendix 3 [file mental-v13-e80765-s003.docx]

**Missingness patterns**

We used the Python package missingno (Boligur, 2018) to visualize missingness patterns in the sample after all data exclusions. Figure 1 shows data availability (missingness) for each individual feature in our study. For instance, here we see that the standard deviation in distance travelled is missing in 9 individuals (and available for 206 individuals). Figure 2 shows how missingness tends to co-occur for feature pairs. For instance, we observe that total distance travelled in kilometers is always missing (available) when mean distance travelled in kilometers is missing (available). Figure 3 digs deeper into this pattern and shows which groups of features tend to be missing within the same individual. Here we see that when one app feature is missing (available), then the other app features tend to be missing (available) as well, and a similar pattern holds for location features. However, there are also location features that form their own subclusters in terms of correlated missingness.


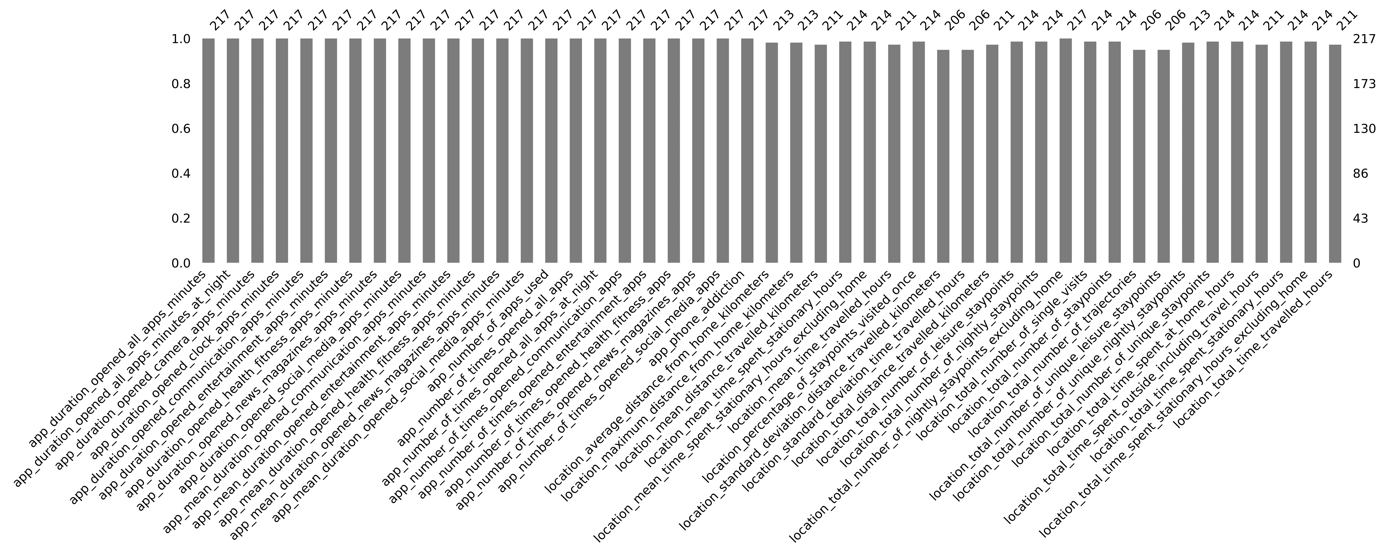


Figure 1: Missingness barplot shows the total number of available observations (y-axis) per feature (x-axis).


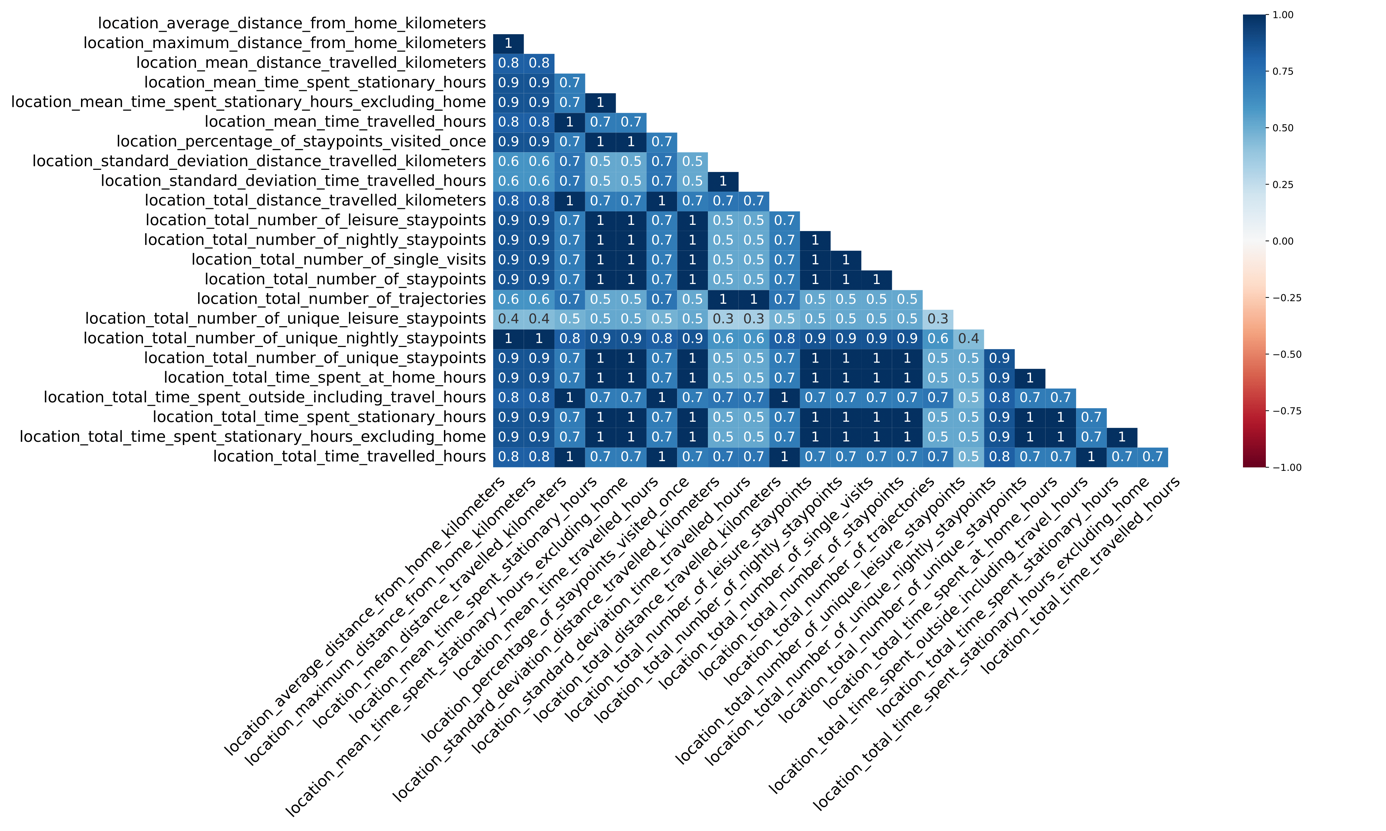


Figure 2: Missingness correlation heatmap shows which variables tend to be missing within the same individual. The stronger the association between the missingness of two features, the stronger their tendency to be missing at the same time, with 1 representing that if feature A is missing, feature B is always missing as well.


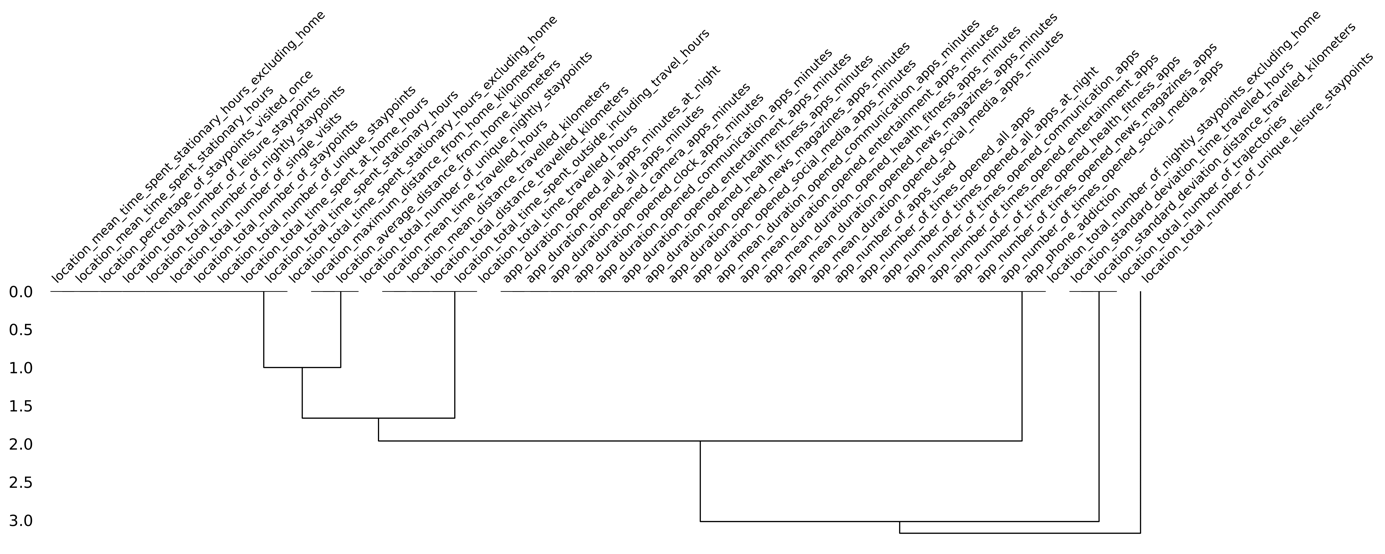


Figure 3: Missingness dendrogram shows how missingness tends to cluster together. Cluster leaves (at the top) perfectly predict one another's presence—one variable might always be empty when another is filled, or they might always both be filled or both empty, and so on. In this figure the dendrogram glues together the variables of

**Bilogur, (2018). Missingno: a missing data visualization suite. Journal of Open Source Software, 3(22), 547, https://doi.org/10.21105/joss.00547**

**Do iOS users differ from Android users?**

Previous research indicates demographic differences between iOS and Android users. Specifically, Android users are more likely to be older, male, and less educated than iOS users (Schoedel et al., 2025). In the sample we analyzed, Android users were slightly older than iOS users, but this difference was not statistically significant after Bonferroni correction for multiple testing (U = 4538, *p* = .389). Similarly, even though Android users had slightly fewer years of education than iOS users (U = 3799.5, *p* = .951) and were more frequently female than male (Chi2=0.0539, *p* = .389), these differences were not statistically significant.

Table 2: Demographic characteristics of Android and iOS users.

|  | Android | iOS |
| --- | --- | --- |
| Age | 53.74 (12.69) | 49.54 (10.93) |
| Years of education | 13.50 (3.11) | 13.17 (4.21) |
| Sex | 66.46% female | 54.17% female |
